# Financial toxicity of cancer care in low- and middle-income countries: a systematic review and meta-analysis

**DOI:** 10.1007/s00520-022-07044-z

**Published:** 2022-04-25

**Authors:** Andrew Donkor, Vivian Della Atuwo-Ampoh, Frederick Yakanu, Eric Torgbenu, Edward Kwabena Ameyaw, Doris Kitson-Mills, Verna Vanderpuye, Kofi Adesi Kyei, Samuel Anim-Sampong, Omar Khader, Jamal Khader

**Affiliations:** 1 Improving Palliative, Aged and Chronic Care Through Clinical Research and Translation (IMPACCT), Faculty of Health, University of Technology Sydney, New South Wales Sydney, Australia; 2 Department of Medical Diagnostics, Faculty of Allied Health Sciences, Kwame Nkrumah University of Science and Technology, Kumasi, Ghana; 3 Department of Medical Imaging, School of Allied Health Sciences, University of Health and Allied Sciences, Ho, Ghana; 4 National Centre for Radiotherapy, Korle-Bu Teaching Hospital, Accra, Ghana; 5 Department of Physiotherapy and Rehabilitation Sciences, University of Health and Allied Sciences, Ho, Ghana; 6 The Australian Centre for Public and Population Health Research (ACPPHR), Faculty of Health, University of Technology Sydney, Sydney, NSW Australia; 7 Department of Radiography, University of Ghana, Accra, Ghana; 8 Faculty of Medicine, University of Jordan, Amman, Jordan; 9 Radiation Oncology Department, King Hussein Cancer Centre, Amman, Jordan

**Keywords:** Cancer, Treatment, Financial toxicity, Low- and middle-income countries

## Abstract

**Introduction:**

The costs associated with cancer diagnosis, treatment and care present enormous financial toxicity. However, evidence of financial toxicity associated with cancer in low- and middle-income countries (LMICs) is scarce.

**Aim:**

To determine the prevalence, determinants and how financial toxicity has been measured among cancer patients in LMICs.

**Methods:**

Four electronic databases were searched to identify studies of any design that reported financial toxicity among cancer patients in LMICs. Random-effects meta-analysis was used to derive the pooled prevalence of financial toxicity. Sub-group analyses were performed according to costs and determinants of financial toxicity.

**Results:**

A total of 31 studies were included in this systematic review and meta-analysis. The pooled prevalence of objective financial toxicity was 56.96% (95% CI, 30.51, 106.32). In sub-group meta-analyses, the objective financial toxicity was higher among cancer patients with household size of more than four (1.17% [95% CI, 1.03, 1.32]; *p* = 0.02; *I*^2^ = 0%), multiple cycles of chemotherapy (1.94% [95% CI, 1.00, 3.75]; *p* = 0.05; *I*^2^ = 43%) and private health facilities (2.87% [95% CI, 1.89, 4.35]; *p* < 0.00001; *I*^2^ = 26%). Included studies hardly focused primarily on subjective measures of financial toxicity, such as material, behavioural and psychosocial. One study reported that 35.4% (*n* = 152 of 429) of cancer patients experienced high subjective financial toxicity.

**Conclusions:**

This study indicates that cancer diagnosis, treatment and care impose high financial toxicity on cancer patients in LMICs. Further rigorous research on cancer-related financial toxicity is needed.

**Supplementary Information:**

The online version contains supplementary material available at 10.1007/s00520-022-07044-z.

## Introduction

New cases and deaths from cancer continue to increase in low- and middle-income countries (LMICs). During the period 2012–2018, the annual new cancer cases increased from 8 million to 9.9 million and cancer deaths increased from 5.3 million to 6.7 million in LMICs [1, 2]. Governments have a responsibility of providing appropriate, accessible and affordable services to the increasing number of cancer patients. However, multiple influential factors, such as unpredictable political climate, inadequately trained cancer care providers, poor coordination and the increasing cost of cancer care, make it difficult to achieve high-quality prevention, early detection, diagnosis, treatment, survivorship and palliative care services [3].

The cost of care is an important barrier to many cancer patients seeking treatment and care. Several LMICs spend about 4 to 7% of their gross domestic product (GDP) on health, with regional differences in patients’ ability and willingness to pay for medical and non-medical care [4]. In most LMICs, there is little or lack of widespread health insurance coverage. Even among patients with health insurance, many are inadequately protected against the costly demands of cancer care because of high costs of insurance, including higher co-payments and increased deductibles. Cancer patients often spend relatively high out-of-pocket for cancer care [5]. The financial support of informal carers is substantial; yet estimates of informal caregiving costs in cancer care have been neglected. Cancer patients and informal caregivers who are often, but not always, family members are vulnerable to losing employment and have a greater risk of personal bankruptcy [6, 7].

There remains a lack of a uniform terminology in the literature to describe the medical and non-medical cancer care costs that result in financial burden for cancer patients and their informal caregivers. A broad definition of financial toxicity was recently proposed as “the possible outcome of perceived subjective financial distress resulting from objective financial burden” [8]. Objective financial burden refers to direct costs and indirect care–related costs while subjective financial distress include material, psychosocial stress, negative emotions and behavioural reactions to cancer care [7, 8]. Terms commonly used interchangeably with financial toxicity include financial or economic difficulty, financial hardship, financial risk and economic stress [9]. Efforts have been made to develop tools for measuring cancer patients’ risk of experiencing financial toxicity, which include COmprehensive Score for Financial Toxicity (COST) [10], Personal Financial Wellness (PFW) Scale [11] and Cancer Survivors’ Unmet Needs (CaSUN) measure [12]. These tools were developed and/or validated with cancer patients from high-income countries (HICs) in mind. The lack of practical guidance and tools that are psychometrically acceptable across settings in LMICs for identifying cancer patients at risk of developing financial toxicity hinders cancer care providers from implementing policies.

A recent systematic review with included studies mostly from HICs identified that cancer patients who were younger, non-white, unmarried, living with dependents and residing in non-metropolitan service areas are more at risk of financial toxicity [13]. There has been proliferation of studies using quantitative design to investigate financial toxicity among cancer patients in LMICs. Hence, it seems timely to conduct a systematic review and meta-analysis to objectively summarise the results to address significant gaps in terms of designing and implementing innovative strategies in LMICs. The study aimed to determine the prevalence, determinants and how financial toxicity has been measured among cancer patients in LMICs, which will be helpful in future studies of financial toxicity.

## Methods

This systematic review followed the Preferred Reporting Items for Systematic Reviews and Meta-Analyses (PRISMA) guideline [14]. It was registered with the international Prospective Register of Systematic Reviews (PROSPERO) (CRD42020207205) [15].

### Eligibility criteria

The inclusion criteria were as follows: primary studies of any design that reported financial toxicity experienced by cancer patients, studies conducted in any country classified as LMIC by the World Bank Group in 2020 (i.e. LMICs are categorised into low-income countries [$1045 or less], low-middle income [$1046 to $4095] and upper-middle-income [$4096 to $12,695]), studies that focused on people with any type of cancer, studies published in peer-reviewed journals and studies published in the English language to capture the current complexity of financial toxicity. Editorials, opinion pieces, comments, letters, reviews and studies focused on high-income settings were excluded.

### Information sources

Four electronic databases were searched, namely Ovid Embase, Ovid MEDLINE® and In-Process & Other Non-Indexed Citations, Cumulative Index to Nursing and Allied Health Literature (CINAHL) and Cochrane Library. A hand search of the reference lists of included studies was performed to supplement the database search.

### Search strategy

Databases were searched on September 7, 2020. The search strategy included terms relating to the following concepts: cancer, cancer patients, delivery of health care, cost of illness, cancer survivors and LMICs. Medical subject headings, keywords and free text terms were combined using “AND” or “OR” Boolean operators. The initial search strategy was developed in MEDLINE (Ovid) (Supplementary Table 1).

### Study selection

Two authors independently screened titles and abstracts of citations retrieved by the search for relevance against the inclusion criteria, and full texts of articles were obtained. Ten per cent of the articles was independently screened by a third author. Disagreements were resolved through discussion.

### Data extraction

An electronic data extraction form was developed, and full-text data extraction was performed by three authors. The extracted data was reviewed and discussed in a team meeting, and disagreements were resolved through consensus. Data extracted included general information, study eligibility, setting, cancer type, study design, data collection, participants, outcome measures and results.

### Quality assessment

Two reviewers assessed the quality of the included studies. Qualitative studies were assessed by using the Joanna Briggs Institute Critical Appraisal Checklist for Qualitative Research [16]. Quantitative studies were assessed according to the appropriate Joanna Briggs Institute Critical Appraisal Checklist, such as cross-sectional studies [17] and cohort studies [18]. Disagreements were resolved by discussion. To enable comparison, each item in the appraisal checklist was rated using a 3-point scale, with “1 = yes, − 1 = no and 0 = not applicable”. The sum was divided by the number of items in the appraisal checklist and multiplied by 100%. The risk of bias scores were categorised as ≥ 80% (low), 60 to 80% (moderate) and < 60% (high).

### Data synthesis and analysis

We used quantitative data to determine the prevalence and determinants of financial toxicity. Meta-analysis was employed for studies that reported quantitative data. A random-effects meta-analysis of odds ratio (OR) was used to calculate pooled data with 95% confidence intervals (CIs). Heterogeneity among studies was estimated using the *I*^2^ index, with values classified as “low heterogeneity” (less than or equal to 25%), “moderate heterogeneity” (26–50%) and “high heterogeneity” (greater than 50%) [19, 20]. Leave-one-out sensitivity analysis was performed to examine whether single studies had a disproportionally excessive influence. Sub-group meta-analyses were conducted to determine the potential sources of heterogeneity. Forest plots were generated. Probability values below 0.05 were considered statistically significant. Data were analysed using Review Manager 5.3.

Qualitative data investigates how certain coping strategies were adopted to address financial toxicity. A narrative synthesis was undertaken for studies that reported qualitative data by comparing similarities and differences across studies [21]. Studies were independently coded by two authors by applying the socio-ecological framework to determine the coping strategies adopted to reduce financial toxicity. Emerging themes were explored and refined, and any discrepancies were resolved through discussion. The socio-ecological framework is suitable to provide a multi-level perspective and structured approach to understanding coping strategies for reducing financial toxicity among cancer patients in LMICs. It is a four-tier framework for organising factors, which then inform corresponding coping strategies [22]. The four levels are individual, relational, community and societal levels. Individual-level factors relate to person characteristics such as age, gender and health conditions. Relational-level factors are defined by direct person-to-person interaction such as family, peer and social support or withdrawal. Community-level factors pertain to workplaces, neighbourhoods, churches and non-governmental/charity organisations. Social-level factors include policies, laws and social and cultural norms [22].

## Results

**Fig. 1:**
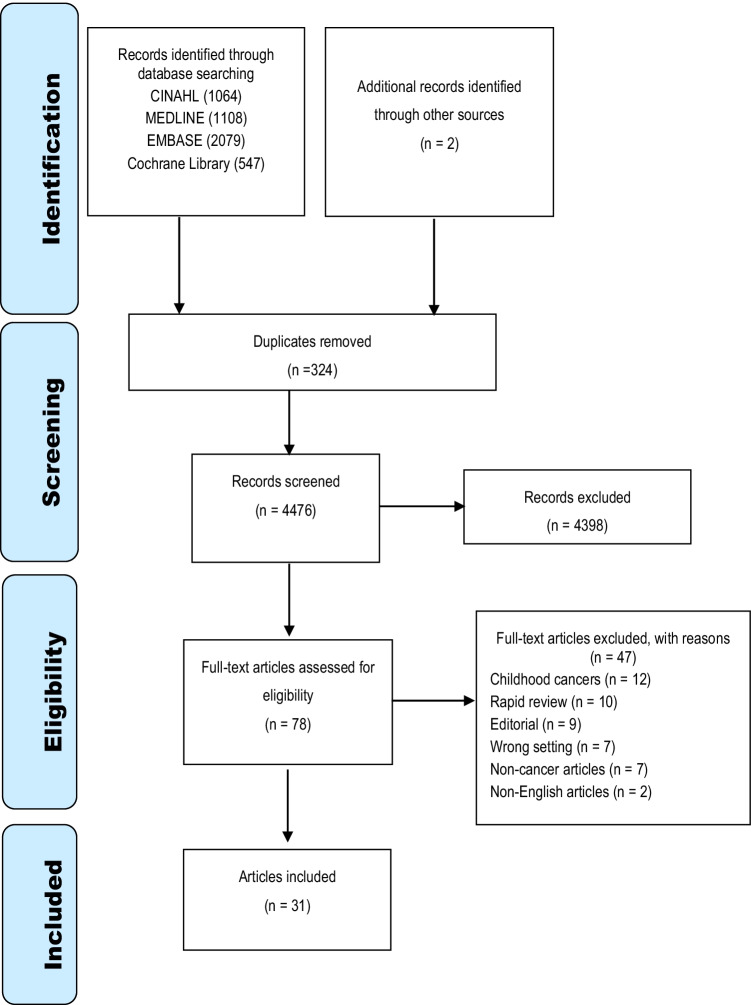
PRISMA flow diagram

The electronic databases searches yielded 4798 articles, with another two identified through hand search. A total of 324 articles were excluded due to duplication. The title and abstract of remaining articles were screened, and 4398 articles were excluded because they did not meet the inclusion criteria. The full text of the remaining 78 articles was then reviewed for eligibility, of which 31 were found to be eligible for inclusion. The PRISMA flow diagram provides detail of the screening process (Fig. 1).

### Characteristics of included studies

Table 1 presents the characteristics of included studies. The 31 studies (30 quantitative and one qualitative) were conducted in four different regions, including Asia (China, *n* = 10; Iran, *n* = 3; Thailand, *n* = 3; Turkey, *n* = 3; Vietnam, *n* = 2; and Malaysia, *n* = 2), Africa (Kenya, *n* = 2; Ethiopia, *n* = 1; and Morocco, *n* = 1), Middle East (Jordan, *n* = 1), South America (Brazil, *n* = 1) and Europe (Serbia, *n* = 1), with a multinational study exploring financial toxicity across Malaysia, Thailand, Indonesia, Philippines, Vietnam, Laos, Cambodia and Myanmar [23]. The quantitative data were based on 14 retrospective cohort studies, 11 cross-sectional studies, four prospective longitudinal and one observational cohort study. One-third of the studies (*n* = 10) were published in 2018 and one-fifth (*n* = 7) in 2019. The total sample size was 120,883, which ranged from 30 to 45,692 participants. Majority of the participants were females (*n* = 65,564). The mean age of the participants was 57.7 ± 7.8 years and ranged from 42 to 72 years. The majority of the studies focused on specific cancer types, such as lung [24–28], breast [29–31], colorectal [32, 33], liver [34], ovarian [35], prostate [36] and stomach [37].

**Table 1:** Characteristics of the included studies

Authors	Year	Aim	Study design/data collection	Country	Sample size	Cancer type	Cancer stage	Gender	Mean age (years)	Tools for measuring financial toxicity	Key results
a. Included quantitative studies											
Ting et al. [38]	2020	To determine the prevalence and associated factors of objective and subjective financial toxicity among urologic cancer patients in Malaysia. Secondly, it investigated the association between financial toxicity and HRQoL	Prospective, cross-sectional/questionnaire	Malaysia	429	Prostate = 366, bladder = 31 and kidney = 32	I = 48, II = 179, III = 85 and IV = 117	M = 414 and F = 15	NR	Used the catastrophic health expenditure tool and Personal Financial Wellness Scale to measure financial toxicity	•Greater objective and subjective financial toxicities were associated with poor HRQoL •Patients attending a private tertiary hospital were more likely to face objective financial toxicity (OR = 258.14, 95% CI = 22.16–3007.58) •Female respondents were more likely to face average to high subjective financial toxicity (OR = 44.88, 95% CI = 4.58–440.12)
Su et al. [39]	2020	To estimate the proportion of Chinese cancer survivors experiencing financial hardship and then examine the relationship between material and behavioural financial hardship	Cross-sectional/questionnaire	China	964	Breast = 398, lung = 140, colorectal = 198 and stomach = 228	NR	M = 366 and F = 598	NR	Material financial problem questions: How much did you or your family borrow or how much debt did you incur because of your cancer, its treatment or the lasting effects of that treatment? Behavioural financial hardship questions: Have you ever forgone cancer treatments because of worrying about the costs? And if so, which cancer treatment is it?	•Almost half of survivors experienced material financial hardship •10% of cancer survivors reported experiencing behavioural financial hardship
Kasahun et al. [40]	2020	To examine the incidence of catastrophic health expenditure and identify associated factors and coping strategies among patients attending cancer treatment services in Addis Ababa, Ethiopia	Cross-sectional/questionnaire	Ethiopia	352	Breast = 130, cervical = 58, colorectal = 46, NPC = 13 and others = 105	NR	M = 94 and F = 258	48 ± 13.2	Structured questionnaire based on WHO Study of Global Ageing and Adult Health Household income and expenditure were measured based on respondents’ self-reported daily or monthly income and expenditure	•74.4% of patients experienced CHE with mean overall expenditure of $2366 per patient •Inpatient services accounted for 2-thirds of the total expenditure with a mean cost of $1584
Zhao et al. [41]	2019	To measure the comprehensive needs of cancer patients and explore the possible factors associated with their needs	Cross-sectional/questionnaires	China	200	General	NR	M = 96 and F = 104	54.87 ± 12.45	Used the comprehensive needs assessment tool (CNAT) in cancer for patients to measure financial burden	•84.5% of the patients had medical insurance •Patients who were younger, female, with low family monthly income, at their own expense, more than 3 years after diagnosis, and with highly educated caregivers had higher score of CNAT (49.13 ± 10.13) •Lowest score of CNAT was the need for physical symptoms (35.12 ± 16.68)
Tekin and Saygili [29]	2019	To determine the annual direct medical costs of all breast cancer patients in Turkey with top-down cost approach	Retrospective cohort/electronic records	Turkey	26,664	Breast	NR	M = 2432 and F = 24,232	NR	Hospital billing system	•Total medical cost of outpatients was $73,534,475.5 •Total medical cost of inpatients was $23,159,274.9 •Total cost of drugs and medical equipment was $14,805,009.2
Rozman et al. [42]	2019	To describe the resource utilisation and costs among cancer patients by cancer localisation and per month of treatment before death	Retrospective cohort/electronic records	Brazil	2985	Lung = 370; colon and rectal = 368; H&N = 353; stomach = 243; breast = 221; female genital tract = 176; oesophagus = 145; prostate = 139; bladder = 94; malignant melanoma = 92; haematological = 90; liver = 87; pancreas = 80; kidney = 71; malignant neoplasm, not otherwise = 65; other sites in the digestive tract = 62; male genital tract = 18; thyroid = 14; others = 146	NR	M = 1629 and F = 1356	64.4 ± 13.46	Hospital billing system	•Average cost per patient was $12,335, ranging from $8269 for patients with pancreatic cancer to $19,395 for patients with brain cancer
Piroozi et al. [43]	2019	To investigate the prevalence as well as the effective factors on facing CHE after the implementation of health transformation plan	Cross-sectional/questionnaire	Iran	161	Oesophageal = 36, stomach = 34, colon and rectum = 67 and others = 24	I = 26, II = 30, III = 37 and IV = 68	M = 152 and F = 9	NR	World Health Survey Questionnaire developed by the WHO	•Lack of supplementary health insurance and low socio-economic status were the significant factors affecting exposure to CHE •The rate of households facing CHE was 72.7%
Moghadam et al. [36]	2019	To investigate the economic burden of prostate cancer patients and their health-related quality of life	Retrospective cohort/questionnaire	Iran	499	Prostate cancer	NR	M = 449	72 ± 9.25	Self-developed questionnaire based on opinions of urology and oncology specialists and experts in the field of economics	•The mean score for HRQoL was 0.62 ± 0.16 for all patients •Chemotherapy patients suffered the worst scores in the physical well-being subscale (0.47 ± 0.24)
Leng et al. [44]	2019	To explore the prevalence, determinants and consequences of CHE among urban and rural end-of-life cancer patients in China	Retrospective cohort/questionnaires	China	792	Lung = 187, intestinal = 53, gastric = 126, liver = 135, oesophagus = 86 and others = 205	NR	M = 539 and F = 253	Urban dwellers = 64.75 Rural dwellers = 64.01	Unvalidated questionnaire that collected information such as demographic characteristics, health services utilisation and end-of-life out-of-pocket payments	•94.3% of urban households and 96.1% of rural households spent 40% or more of their monthly income as out-of-pocket cancer health care expenditure •The poorer the household, the higher the prevalence of catastrophic health expenditure •Health insurance did not adequately compensate for CHE
Bhoo-Pathy et al. [45]	2019	To examine the incidence, cost drivers and factors associated with financial toxicity after cancer in an upper–middle-income country with universal health coverage	Prospective longitudinal cohort/cost dairies and questionnaire	Malaysia	1294	GI = 345, respiratory = 62, breast = 424, female reproductive = 81, urogenital = 14, hematologic = 278 and other = 90	I = 59, II = 153, III = 103, IV = 170 and unknown = 531	M = 470 and F = 824	Median = 53	Unvalidated questionnaire that collected cost data on conventional medical care, traditional and complementary medical care and goods and services related to cancer care (transportation, meals, lodging, parking, childcare and personal items directly incurred by patients and not reimbursed by insurance	•Overall incidence of financial toxicity among the cancer survivors at 1 year was 51% (*n* = 665), ranging from 33% in MOH hospitals to 65% in the public university hospital and 72% in the private hospitals •Low-income status, type of hospital and lack of health insurance were strong predictors of financial toxicity •Payments for conventional medical care made up 39% of the total OOP costs borne by the affected households
Zheng et al. [46]	2018	To evaluate the medical economic burden, including total current curative expenditure and CHE on cancer in Liaoning Province, China	Retrospective cohort/medical records and questionnaire	China	1344	General	NR	M = 775 and F = 569	NR	Questionnaire surveys among cancer patients, which collected information of demographic characteristics, household income and expenditure, medical expenses and compensation	•Incidence of CHE was 42.78%. Influencing factors were length of stay, type of health insurance and location of household •Among the households, those with oesophageal cancer patients were most likely to experience CHE of which the incidence rates were 60.29%, 57.89% and 46.89%
Sun et al. [37]	2018	To examine the costs of the first course treatments in Chinese patients with stomach cancer and the associated trends	Retrospective cohort/medical records	China	14,692	Stomach	I = 2357, II = 2590, III = 3452, IV = 4838 and unknown = 1060	M = 10,092 and F = 4205	58.1 ± 12.6	Hospital information system that collected medical expenses for cancer treatments including payments (both of out-of-pocket payments and payments by insurance plans) of each patient for admissions and outpatients from the first admission date to the last discharge date	•Average medical expenses of the first course treatments were about $6851 •Contributing factors included long stay in hospital and an increased number of episodes of care
Saengow et al. [32]	2018	To determine the willingness to pay for faecal immunochemical test and colonoscopy and examine an effect of proposed co-payment on uptake rates	Cross-sectional/questionnaire	Thailand	437	Colorectal		M = 183 and F = 254	58.4 ± NR	Willingness to pay for colorectal cancer screening questionnaire	•Less than half of participants were willing to pay for colonoscopy •Presence of companion, female and family history of cancer were influential factors
Qiu et al. [34]	2018	To understand the medical expenditure for liver cancer during 2002–2011 in urban areas of China	Retrospective cohort/questionnaire	China	12,342	Liver	I = 905, II = 3089, III = 4683, IV = 2556 and unknown = 1109	M = 9638 and F = 2704	54.91 ± 12.29	Individual case–based medical care cost records, with data such as demographic, diagnostic information and detailed expenditure information relating to different types of service including registration, ward bed, diagnosis, examination, treatment, surgery, laboratory, nursing and drugs	•Pharmaceuticals accounted for the biggest part of the medical expenditure, and it rose from 48.01 to 52.96%
Perin et al. [24]	2018	To assess the hospital costs of diagnosing and treating patients with stage IIIB and IV non-small cell lung cancer	Retrospective cohort/electronic records	Serbia	187	Lung	IIIB = 69 and IV = 118	M = 137 and F = 50	NR	Extracted resources and procedures from the integrated hospital information system to estimate the cost for each patient	•The average hospital cost per patient was $3309.40 •37% of the hospital cost was due to medication
Owenga and Nyambedha [47]	2018	To assess the financial challenges and sources of financial assistance for cervical cancer patients	Cross-sectional/questionnaire	Kenya	334	Cervical	I = 52, II = 40, III = 63 and IV = 129	F = 334	NR	Self-designed questionnaire that collected information such as socio-demographics and health history, financial challenges of cervical cancer patients, patient care and information needs and spiritual needs	•Financial challenges were costs of medication 291 (87%), cost of travel 281 (84%) and cost of diagnostic tests 250 (75%) •13% of patients received assistance from charity organisation, 27% received assistance from friends, 9% received assistance from colleagues, 10% received assistance from relatives and 10% received assistance from church
Nguyen, et al. [48]	2018	To estimate the medical costs for the treatment of cervical cancer patients	Retrospective cohort/medical records and expert discussion	Vietnam	52 patients and 10 experts	Cervical	NR	F = 62	NR	Reviewed medical records of 52 patients with cervical cancers to document the medical procedures, types and quantity of resources needed for the service	•The unit costs for precancer services fluctuated from $18.26 to $33.31 •The main cost driver of radical hysterectomy and radiotherapy was the staff payments (59%)
Liao et al. [30]	2018	To assess the economic burden of breast cancer (BC) diagnosis and treatment in China through a multicentre cross-sectional study and to obtain a theoretical evidence for policy-making	Cross-sectional/questionnaire	China	2746	Breast	I = 546, II = 1236, III = 603, IV = 285 and unknown = 76	F = 2746	49.6 ± 10.0	Questionnaire comprising of demographic characteristics, clinical information and relative expenditure information (dates of diagnosis and treatment, all medical expenditure [self-pay and healthcare costs], non-medical expenditure [transportation, accommodation, meals, nutrition and employee escort fees])	•Overall average expenditure was $8450 (medical expenditure: $7527; non-medical expenditure: $922) •Average loss of time was $1529
Chen et al. [25]	2018	To examine the effect of financial burden, using objective and subjective indicators, on the HRQoL in lung cancer patients	Cross-sectional/medical records and questionnaires	China	227	Lung	I = 12, II = 28, III = 57 and IV = 130	M = 159 and F = 68	59.48 ± 9.42	Financial information was collected using 4 questions: How much did you pay for the medical expense last month? (direct medical costs) How much did you spend on the disease-related expenses other than medical expenses, such as buying health supplements, last month? (direct nonmedical costs) What proportion of your annual household income do you spend on healthcare annually? (healthcare cost-to-income ratio) Have your disease and treatment caused you and your family financial difficulty? (perceived financial difficulty)	•Financial difficulty was perceived in 83.7% of the participants •Mean direct medical costs was $2518.83 with a median of $1515.01 and a range from $60.60 to $18,180.17 •27.3% reported that the healthcare cost-to-income ratio was less than 40%
Atieno et al. [49]	2018	To evaluate the economic burden of treating cancer patients	Cross-sectional/questionnaire	Kenya	412	General	NR	M = 152 and F = 261	NR	The self-designed questionnaire collected information such as patient demographics, medicines prescribed and their costs, cost of radiologic tests, costs of laboratory tests, any surgery and associated costs and quantity and costs of any medical devices used	•Patients on chemotherapy alone cost an average of $1364.3, surgery cost $1265.6, radiotherapy $1175.1 and combination of all 3, $3291.8 per patient
Zhuyan et al. [35]	2017	To assess the association of financial status and QoL among Chinese women actively undergoing chemotherapy for recurrent ovarian cancer	Prospective, longitudinal cohort/medical records, questionnaires	China	123	Ovarian	NR	F = 123	53.4 ± 9.3	Reviewed medical records to document information such as demographic and clinical data (age, marital status, education, occupation, type of insurance, financial status and number of recurrences and intervals between recurrences) Financial status was based on self-reported annual family income minus expenses Quality of life was evaluated using the simplified Chinese version (3.0) of the European Organization for Research and Treatment of Cancer (EORCT) 30-Item Core Quality of Life Questionnaire (QLQ-C30) and the simplified Chinese version of the QLQ-OV28 questionnaire which is specific to ovarian cancer	•Patients with low financial status had a significantly higher risk of deteriorating HRQoL in physical functioning (*p* = 0.001), role functioning (*p* = 0.0140), emotional functioning (*p* = 0.021), pain (*p* = 0.010) and financial difficulties (*p* = 0.003)
Wenhui, Shenglan [50]	2017	To analyse the health services utilisation and financial burden of insured cancer patients and identify the gaps of financial protection provided by insurance in urban China	Retrospective cohort/medical records	China	Shanghai = 600 Beijing = 600 Fuzhou = 600 Chongqing = 608	General	NR	Shanghai M = 289 and F = 311 Beijing M = 325 and F = 275 Fuzhou M = 311 and F = 289 Chongqing M = 346 and F = 262	Shanghai = 63.5 ± 12.4 Beijing = 63.2 ± 13.5 Fuzhou = 67.1 ± 10.0 Chongqing = 67.4 ± 13.7	Hospital records of participants were extracted. Average total expense per visit, average out-of-pocket payments and average reimbursement rate were analysed	•The average OOP as the proportion of household’s capacity to pay was 87.3% (Chongqing), 66.0% (Fuzhou), 33.7% (Beijing) and 19.6% (Shanghai)
Thongprasert et al. [26]	2015	To evaluate the patient and public willingness to pay for a quality-adjusted life year for lung cancer treatments using Thailand as an example	Cross-sectional/questionnaire	Thailand	150	Lung	I–II = 5 and III–IV = 145	M = 78 and F = 72	60.9 ± 10.40	The questionnaire collected information such as socio-demographic data, respondents’ health status/utility and willingness to pay	•Patients’ willingness to pay was associated with quality of life, financial difficulties, health insurance, diarrhoea and wealth
The Action Study Group et al. [23]	2015	To determine, in this region, in patients with a first-time diagnosis of cancer and in whom surgery was specified in their initial treatment plans, the incidence of financial catastrophe owing to out-of-pocket payments for treatment, treatment discontinuation (as defined by whether such patients proceed to hospitalisation by 3 months), and mortality, as well as the factors associated with such outcomes	Prospective, longitudinal cohort/questionnaire	8-country (Malaysia, Thailand, Indonesia, Philippines, Vietnam, Laos, Cambodia and Myanmar)	4584	Breast = 1667; mouth and pharynx = 388; stomach = 227; colon and rectum = 622; trachea, bronchus and lung = 116; cervix = 341; uterus = 135; ovary = 179; and others = 779	I = 415, II = 1181, III = 944 and IV = 483	M = 1300 and F = 3284	51.3 ± 12.4	The questionnaire collected information such as participants’ age, sex, marital status, country of residence, highest level of education attained, employment status and recent experience of economic hardship whether in the previous 12 months they were unable to make any necessary household payments or needed assistance to do so, annual household income and health insurance status	•31% of participants incurred financial catastrophe. Women had greater odds of financial catastrophe than men (OR, 1.35; 95% CI, 1.05–1.74)
Lkhoyaali et al. [51]	2015	To assess the social, psychological, behavioural and economic impact on patient’s family caregivers	Prospective cohort/questionnaire	Morocco	150	Lung, breast and lymphoma	NR	M = 61 and F = 89	44.7	Participants’ demographics, disease characteristics and social, economic and psychological features were collected with a questionnaire. Psychological impact was assessed using Diagnostic and Statistical Manual of Mental Disorders	•Economic resources were exceeded in 78.7%. 56% used banking credits and sold properties. Work lay-off was recorded in 54%
Ak et al. [27]	2015	To evaluate the relationship between cost according to treatment type and prognosis in malignant PI	Retrospective cohort/medical records	Turkey	275	PM	I–II = 50, III–IV = 221 and unknown = 4	M = 146 and F = 129	63.2 ± 11.2	Medical records of participants were reviewed. Direct medical costs were estimated as the sum of hospital bills attributed to the disease. The phases of care were divided into 3 periods as diagnosis, treatment and terminal phase in chemotherapy and multimodality groups	•Factors affecting the cost were histology, treatment type, received second- and third-line chemotherapy and number of hospitalisations
Nguyen et al. [31]	2013	To estimate the direct medical cost of a 5-year treatment course for women with primary breast cancer in Central Vietnam	Retrospective cohort/medical records	Vietnam	129	Breast	I = 9, II = 73, III = 35 and V = 12	F = 129	51 ± 9.5	Medical records were reviewed to obtain personal information (e.g. name, age, home address), date of admission, diagnosis and stage, treatment regimes, itemized invoices and health insurance participation. Unit costs for treatments received over the study period were acquired from the hospital’s finance department	•Total direct medical cost for a 5-year treatment course was estimated at $975 per patient (range: $11.7–$3955)
Nazer et al. [52]	2013	To describe the drug utilisation pattern and drug cost in the treatment of cancer patients with severe sepsis and septic	Retrospective cohort/electronic records	Jordan	116	General	NR	M = 65 and F = 51	51.7 ± 14.8	Electronic medical records were reviewed to determine the total number of medications prescribed, the type of medications, and the cost of each medication	•Mean number of medications prescribed per patient were 11.7 (SD ± 4.7)
Chindaprasirt et al. [33]	2012	To identify admission rates and healthcare cost of colorectal cancer	Retrospective cohort/medical records	Thailand	45,692	Colorectal	NR	M = 24,068 and F = 21,624	NR	Medical records were reviewed to collect data such as age, gender, level of hospital, regions of hospital, admission rate and hospital costs	•Colorectal cancer contributed to 98.5 per 100,000 adult persons admission rates •Average hospital charge per admission were $1360.06
Edis and Karlikaya [28]	2007	To evaluate the individual and societal costs of lung cancer derived from our patient representative	Observational cohort/medical records	Turkey	103	Lung	NR	M = 98 and F = 5	64 ± 9.3	Hospital billing system were reviewed to obtained direct medical costs and additional medical costs associated with the diagnosis and treatment of lung cancer	•Average survival was 6.8 months •Average cost per patient was $5.480 ± 4.088 •Direct medical cost was $5.471 ± 4.091
b.*Included qualitative studies*											
Moradian et al. [53]	2012	To explore, through qualitative semi-structured interviews, Iranian cancer patients’ needs	Qualitative, descriptive/semi-structured interviews	Iran	30	Breast = 7, GI = 5, bladder = 2; testis = 2, lung = 2, sarcoma = 2 and other = 10	I = 8, II = 15 and III-IV = 7	M = 11 and F = 19	42 years and ranged between 19 and 59 years	Semi-structured interviews with interview guide focused on the main theme: ‘patients need to express feelings about the disease, impact of cancer on their daily life and their experiences of existing services’	•Major themes: financial issues (cost of treatment and interference with their ability to work), psychosocial issues (social and significant others support, distress and fear from future) and care satisfaction (accessing information and nursing care)

*NR* not reported, *CHE* catastrophic health expenditure, *HRQoL* health-related quality of life, *OOP* out-of-pocket, *H&N* head and neck, *GI* gastrointestinal, *PM* pleural mesothelioma, *M* male, *F* female.

### Prevalence of objective financial toxicity

**Fig. 2:**
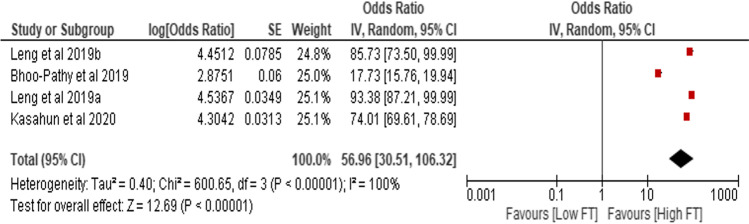
Random-effects meta-analysis of studies that reported the prevalence of financial toxicity

Three studies provided the prevalence estimates of objective financial toxicity [40, 44, 45] enabling a meta-analysis. The pooled prevalence of objective financial toxicity was 56.96% (95% CI, 30.51, 106.32) (see Fig. 2). The random-effects meta-analysis showed that the pooled prevalence of objective financial toxicity among cancer patients varied from 17.73% (95% CI, 15.76, 19.94) to 93.38% (95% CI, 87.21, 99.99) in any cancer type after separating the data on rural and urban in one study [44]. Rural dwellers had a substantially higher prevalence of objective financial toxicity estimates (93.38% [95% CI, 87.21, 99.99]). However, the heterogeneity in the ratio of prevalence was extremely high (*I*^2^ = 100%). Objective financial toxicity was categorised into direct medical costs, direct non-medical costs and indirect costs.

### Direct medical costs

Table 2 presents the results of the mean estimates of cancer care costs using random-effects meta-analysis and sub-group meta-analysis. Direct medical costs were categorised into seven cost items: consultation; diagnosis; treatment, including surgery, radiotherapy, chemotherapy, hormone therapy, combined modalities and palliative/supportive care; inpatient care; outpatient care; and follow-up care. In total, 11 studies presented data on mean direct medical costs [25, 27–29, 31, 36, 37, 40, 42, 49, 52]. Overall mean direct medical costs were $2740.18, which ranged from $1953.62 to $3526.74 per cancer patient. Components of the overall mean direct medical costs included $2366.00 (95% CI, 1920.76, 2811.24) in any cancer type, $1902.95 (95% CI, $ − 655.85, $4461.74) in lung cancer, $4961.80 (95% CI, $4892.80, $5030.80) in stomach cancer, $91.60 (95% CI, $72.87, 110.33) in breast cancer and $6141.30 (95% CI, $5717.88, $6564.72) in prostate cancer, with GDP per capita ranging from $858 in Ethiopia to $10,262 in China.

**Table 2:** Mean estimates of cancer care costs using random-effects meta-analysis and sub-group meta-analysis

Cost variable	Cancer type	No. of articles	Mean (95% CI)	I^2 ^(%)	P value	GDP per capita*	Ratio of cost range as GDP per capita**	Reference
Overall medical costs	Any cancer type	1	$2366.00 ($1920.76, $2811.24)		< 0.00001	$858	223.86–327.65	[37]
	Lung cancer	1	$3199.25 ($3120.88, $3277.62)		< 0.00001	$9042	34.52–36.25	[23]
		1	$2518.83 ($1837.99, $3199.67)		< 0.00001	$10,262	17.91–31.18	[21]
		1	$5.48 ($4.68, $6.28)		< 0.00001	$9042	0.05–0.07	[24]
	Sub-total	*3*	$1902.95 ($ − 655.85, 4461.74)	100	0.14			
	Stomach cancer	1	$4961.80 ($4892.80, $5030.80)		< 0.00001	$10,262	47.67–49.02	[33]
	Breast cancer	1	$91.60 ($72.87, $110.33)		< 0.00001	$9042	0.81–1.22	[25]
	Prostate cancer	1	$6141.30 ($5717.88, $6564.72)		< 0.00001	$5506	103.85–119.23	[32]
	Total	**7**	$2740.18 ($1953.62, $3526.74)	100	< 0.00001	**–**	**–**	
Consultation	Any cancer type	1	$35.70 (32.23, 39.17)		< 0.00001	$1817	1.77–2.16	[40]
Diagnosis	Any cancer type	1	$138.90 ($126.59, $151.21)		< 0.00001	$1817	6.97–8.32	[40]
	Breast cancer	1	$16.02 ($15.12, $16.92)		< 0.00001	$2715	0.56–0.62	[27]
	Prostate cancer	1	$205.80 ($168.32, $243.28)		< 0.00001	$5506	3.06–4.42	[32]
	Total	3	$119.02 ($13.71, $224.33)	100	0.03	**–**	**–**	
Surgery	Any cancer type	1	$1268.30 ($1098.57, $1438.03)		< 0.00001	$1817	60.46–79.14	[40]
	Breast cancer	1	$82.35 ($76.86, $87.84)		< 0.00001	$2715	2.83–3.24	[27]
	Prostate cancer	1	$3709.50 ($3396.01, $4022.99)		< 0.00001	$5506	61.68–73.07	[32]
	Total	3	$1678.80 ($62.39, $3295.20)	100	0.04	**–**	**–**	
Radiotherapy	Breast cancer	1	$22.87 ($16.03, $29.71)		< 0.00001	$2715	0.59–1.09	[27]
	Prostate cancer	1	$8242.60 ($7963.29, $8521.91)		< 0.00001	$5506	144.63–154.77	[32]
	Total	2	$4131.50 ($ − 3923.69, $12,186.69)	100	0.31			
Chemotherapy	Any cancer type	1	$1372.50 ($1050.30, $1694.70)		< 0.00001	$1817	57.80–93.27	[40]
	Lung cancer	1	$10,540.00 ($6615.09, $14,464.91)		< 0.00001	$9042	73.16–159.97	[23]
	Breast cancer	1	$476.48 ($346.57, $606.39)		< 0.00001	$2715	12.77–22.33	[27]
	Prostate cancer	1	$14,181.30 ($13,803.62, $14,558.98)		< 0.00001	$5506	250.70–264.42	[32]
	Total	4	$6555.98 ($97.19, $13,014.76)	100	0.05	**–**	**–**	
Hormone therapy	Breast cancer	1	$4.25 ($1.63, $6.87)		0.001	$2715	0.06–0.25	[27]
	Prostate cancer	1	$2940.40 ($2786.29, $3094.51)		< 0.00001	$5506	50.60–56.20	[32]
	Total	2	$1471.27 ($ − 1406.10, $4348.65)	100	0.32	**–**	**–**	
Surgery + radiotherapy	Any cancer type	1	$1749.35 ($1257.90, $2240.80)		< 0.00001	$1817	69.23–123.32	[40]
Surgery + chemotherapy + radiotherapy	Any cancer type	1	$2547.75 ($1532.40, $3563.10)		< 0.00001	$1817	84.34–196.10	[40]
	Lung cancer	1	$17,210.25 ($17,176.28, $17,244.22)		< 0.00001	$9042	189.96–190.71	[23]
	Total	2	$9888.14 ($ − 4480.83, $24,257.12)	100	0.18	**–**	**–**	
Palliative/supportive care	Any cancer type	1	$976.65 ($749.60, $1203.70)		< 0.00001	$1817	41.25–66.25	[40]
		1	$12,327.00 ($11,803.00, $12,851.00)		< 0.00001	$8717	135.40–147.42	[49]
	Sub-total	2	$6649.27 ($ − 4473.87, $17,772.40)	100	0.24	*–*	*–*	
	Lung cancer	1	$1897.00 ($1849.16, $1944.84)		< 0.00001	$9042	20.45–21.51	[23]
	Breast cancer	1	$4.50 ($2.02, $6.98)		0.0004	$2715	0.07–0.26	[27]
	Total	4	$3741.28 ($2241.19, $5241.37)	100	< 0.00001	**–**	**–**	
Inpatient care	Any cancer type	1	275.10 (241.87, 308.33)		< 0.00001	$1817	13.31–16.97	[40]
		1	1584.00 (1193.76, 1974.24)		< 0.00001	$858	139.13–230.10	[37]
	Sub-total	2	$914.52 ($ − 367.84, $2196.88)	98	0.16			
	Breast cancer	1	$26.38 ($24.28, $28.48)		< 0.00001	$2715	0.89–1.05	[27]
	Total	3	$436.51 ($190.65, $682.36)	99	0.0005	**–**	**–**	
Outpatient care	Any cancer type	1	$782.00 ($638.85, $925.15)		< 0.00001	$858	74.46–107.83	[37]
	Breast cancer	1	$591.60 ($572.87, $610.33)		< 0.00001	$9042	6.34–6.75	[25]
	Total	2	$673.03 ($488.40, $857.66)	85	< 0.00001			
Follow-up care	Breast cancer	1	$356.24 ($311.36, $401.12)		< 0.00001	$2715	11.47–14.77	[27]
Indirect costs	Lung cancer	1	$17.34 ($11.87, $22.80)		< 0.00001	$9042	0.13–0.25	[24]
	Prostate cancer	1	$4873.93 ($3604.88, $6142.98)		< 0.00001	$5506	65.47–111.57	[32]
	Total	2	$2402.47 ($ − 2356.15, $7161.09)	98	0.32	**–**	**–**	
Direct non-medical	Lung cancer	1	$334.00 ($333.74, $334.26)		< 0.00001	$9042	3.69–3.70	[24]
Informal care	Prostate cancer	1	$2454.70 ($2171.84, $2737.56)		< 0.0001	$5506	39.44–49.72	[32]

*CI* confidence interval, *GDP* gross domestic product. *The 2019 data. **The ratio of cost of care to GDP per capita was computed by dividing the mean cost range by the GDP per capita and multiplying by 100.

Three studies reported data on diagnostic costs [31, 36, 49]. Expressed as random-effects estimates, mean diagnosis costs were higher for any cancer type ($138.90 [95% CI, $126.59, $151.21]; *p* < 0.00001), as well as breast cancer in women ($16.02 [95% CI, $15.12, $16.92]; *p* < 0.00001) and prostate cancer in men ($205.80 [95% CI, $168.32, $243.28]; *p* < 0.00001). Consultation costs significantly favoured higher medical costs (*p* < 0.00001) [49]. The ratio of consultation costs to GDP per capita ranged from 1.77 to 2.16 in Kenya.

Costs of surgery were measured in three studies from Kenya [49], Vietnam [31] and Iran [36] with GDP per capita ranging from $1817 to $5506. The pooled mean costs of surgery were $1678.80 (95% CI, $62.39, $3295.20; *p* = 0.04; *I*^2^ = 100%), which varied greatly from breast cancer ($82.35 [95% CI, $76.86, $87.84]; *p* < 0.00001) to prostate cancer ($3709.50 [95% CI, $3396.01, $4022.99]; *p* < 0.00001). On the other hand, data regarding overall mean costs of radiotherapy were available in two studies [31, 36]. A non-significant increase in total mean costs of radiotherapy favouring low costs burden was observed ($4131.50 [95% CI, $ − 3923.69, $12,186.69]; *p* = 0.31; *I*^2^ = 100%), with higher heterogeneity. The ratio of radiotherapy costs to GDP per capita ranged from 0.59 in Vietnam to 154.78 in Iran.

The sub-group meta-analysis of the total costs of chemotherapy favouring high financial toxicity was observed ($6555.98 [95% CI, $ − 97.19, $13,014.76]; *p* = 0.05; *I*^2^ = 100%), showing increased mean costs from $476.48 per breast cancer patients, $1372.50 per any cancer type, $10,540.00 per lung cancer patient, to $14,181.30 per prostate cancer patient. Two studies presented data on mean costs of combined surgery, chemotherapy and radiotherapy [27, 49], with total costs of $9888.14 (95% CI, $ − 4480.83, $24,257.12) and a substantial heterogeneity (*I*^2^ = 100%). One study reported that combined surgery and radiotherapy for any cancer type resulted in even higher associated direct medical costs ($1749.35 [95% CI, $1257.90, $2240.80]; *p* < 0.00001) [49].

Mean costs of palliative care were measured in four studies from Kenya [49], Vietnam [31], Brazil [42] and Turkey [27] with GDP per capita ranging from $1817 to $9042. The random-effects meta-analysis estimated direct medical costs attributed to palliative care as $3741.28 (95% CI, $2241.19, $5241.37). Also, two studies conducted in Ethiopia [40] and Turkey [29] reported data on costs of outpatient care, which was significantly associated with higher financial burden ($673.03 [95% CI, $488.40, $857.66]; *p* < 0.00001; *I*^2^ = 85%). One study from Vietnam [31] with GDP per capita of $2715 reported costs of follow-up care in breast cancer patients as $356.24 ranging between $311.36 and $401.12 per patient.

### Direct non-medical costs

The total direct non-medical costs as reported by one study from Turkey were $334.00 (95% CI, $333.74, $334.26) per lung cancer patient [28]. Direct non-medical costs were observed to be significant (*p* < 0.00001). Components of the direct non-medical costs included disease-related transfer, accommodation and informal and transportation costs. It was observed that mean transportation costs ($162.00 [95% CI, $125.307, $198.693]; *p* < 0.00001) were responsible for 48% of the total direct non-medical costs incurred by lung cancer patients [28]. Also, informal costs were associated with significantly higher direct non-medical costs among prostate cancer patients, with mean costs of $2454.70 ranging between $2171.84 and $2737.56 (*p* < 0.0001) [36]. The ratio of informal costs to GDP per capita ranged from 39.44 to 49.72 in Iran.

### Indirect costs

Two studies conducted in Iran and Turkey with GDP per capita ranging from $5506 to $9042 reported quantitative data on indirect non-medical costs [28, 36]. The overall pooled mean indirect costs were $2402.47 (95% CI, $ − 2356.15, $7161.09), with $17.34 (95% CI, $11.87, $22.80) per lung cancer patient and $4873.93 (95% CI, $3604.88, $6142.98) per prostate cancer patient. However, there was high heterogeneity (*I*^2^ = 98%).

### Prevalence of subjective financial toxicity

Included studies hardly focused primarily on subjective measures of financial toxicity, such as material, behavioural and psychosocial. We were unable to provide pooled prevalence of subjective financial toxicity because only one study provided prevalence estimate. The study reported that 35.4% (*n* = 152 of 429), 11.9% (*n* = 51 of 429) and 52.7% (*n* = 226 of 429) of cancer patients experienced high, average and low subjective financial toxicity, respectively [38]. Psychosocial issues identified in one qualitative study were anxiety and social relationship disruption through conflict and criticism [53].

**Fig. 3:**
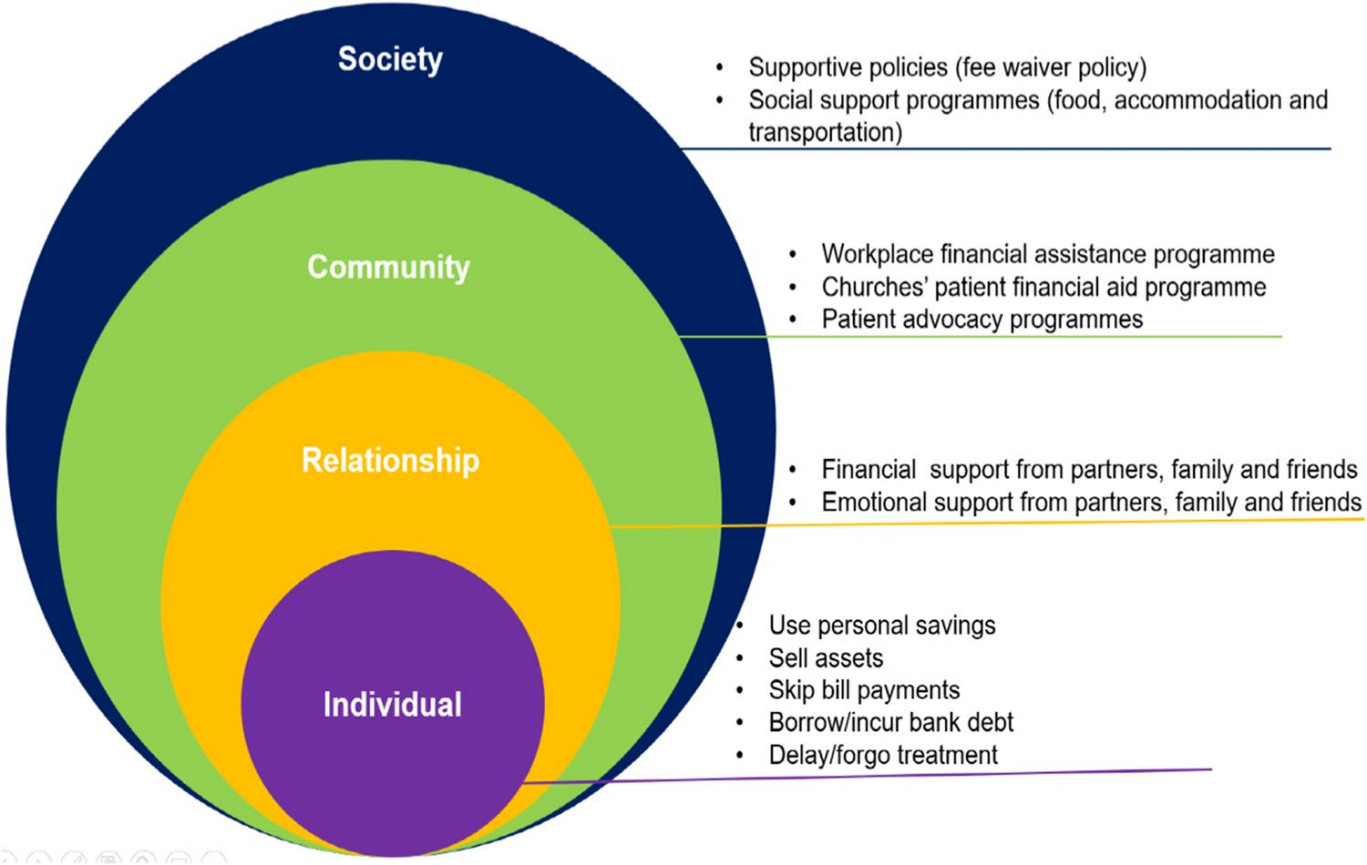
Coping strategies for reducing financial toxicity

Three studies highlighted coping behaviours at the individual level, which included using personal savings, selling assets, skipping bill payments, borrowing or incurring bank debt and delaying/forgoing treatment [40, 47, 51]. Two studies identified coping behaviours at the relational level, such as receiving financial support from family and friends and emotional support from partners, friends and family members [40, 53]. The major coping behaviour at the community level was seeking financial assistance from workplaces, neighbourhoods, churches and non-governmental/charity organisations to cover the financial toxicity imposed on cancer patients and their household [40, 54]. There were two main coping behaviours at the social level, which included creating supportive policies (e.g. a waiver to help cancer patients offset their medical bills) and promoting a pleasant social support environment, such as food, accommodation and transport for treatment programme [53, 54] (see Fig. 3).

### Determinants of objective financial toxicity

**Fig. 4:**
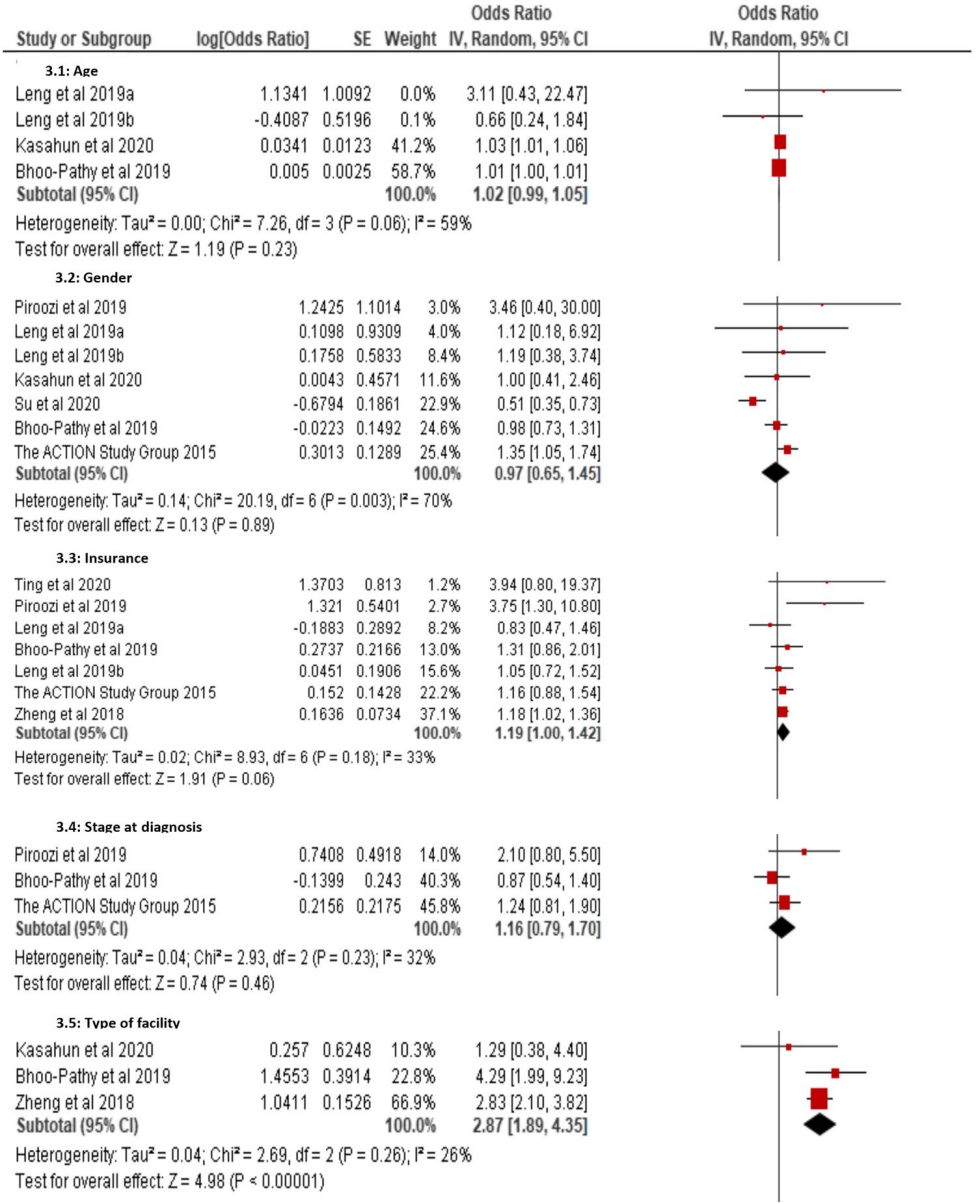

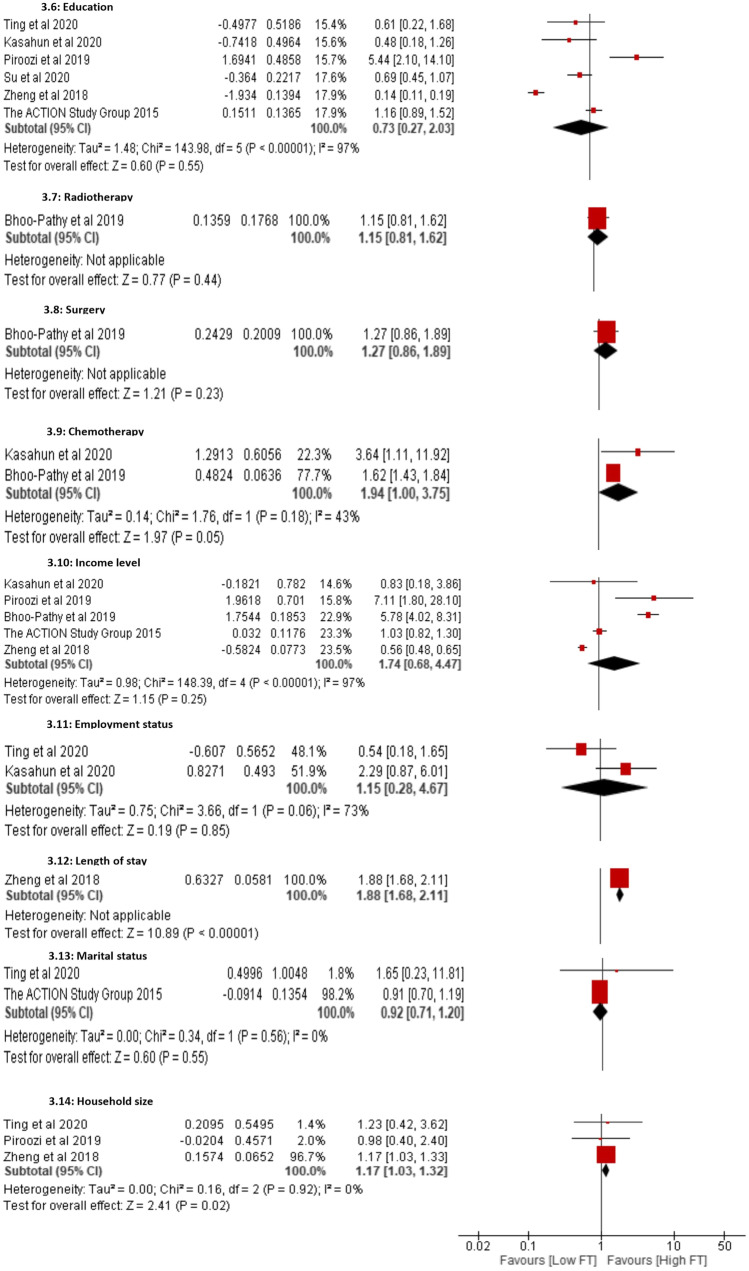
Forest plot showing determinants of objective financial toxicity

It was challenging to perform a meta-analysis of the factors associated with subjective financial toxicity because the instruments and domains differed across studies. Figure 4 presents pooled estimates of the determinants of objective financial toxicity. The sub-group meta-analyses showed that cancer patients with a household size of more than four were associated with a significant increase in objective financial toxicity (1.17% [95% CI, 1.03, 1.32]; *p* = 0.02; *I*^2^ = 0%). There was no significant heterogeneity among the three included studies [38, 43, 46]. The meta-analysis revealed that cancer patients who received more than six cycles of chemotherapy were almost two times more likely to experience high financial toxicity (1.94% [95% CI, 1.00, 3.75]; *p* = 0.05; *I*^2^ = 43%). In three of the included studies [40, 45, 46], it was observed that cancer patients who attended private health facilities during the course of their disease were statistically associated with high-level financial toxicity (2.87% [95% CI, 1.89, 4.35]; *p* < 0.00001; *I*^2^ = 26%). One study indicated that prolonged length of hospital stay was significantly related to cancer patients encountering higher objective financial toxicity (1.88% [95% CI, 1.68, 2.11]; *p* < 0.00001) [46].

Using data from six studies [23, 38, 43–46], the pooled estimate for health insurance as a determinant of objective financial toxicity among cancer patients was not a significant factor (1.19% [95% CI, 1.00, 1.42]; *p* = 0.06; *I*^2^ = 33%). However, according to the leave-one-out sensitivity analysis, the random-effects meta-analysis showed that not having health insurance was a significant risk factor for exposure to objective financial toxicity (1.29% [95% CI, 1.03, 1.61]; *p* < 0.03; *I*^2^ = 42%) when removing one study from China [44] from the pooled analysis. The sub-group meta-analyses indicate no statistically significant association with cancer-related objective financial toxicity by gender (0.97% [95% CI, 0.65, 1.45]; *p* = 0.89; *I*^2^ = 70%), stage at diagnosis (1.16% [95% CI, 0.79, 1.70]; *p* = 0.46; *I*^2^ = 32%), level of education (0.73% [95% CI, 0.27, 2.03]; *p* = 0.55; *I*^2^ = 97%) or income level (1.74% [95% CI, 0.68, 4.47]; *p* = 0.25; *I*^2^ = 97%).

### Measuring financial toxicity

Over one-third of the studies used unvalidated questionnaires to measure the financial toxicity related to cancer care [23, 25, 26, 30, 35, 36, 39, 44, 45, 51, 54]. Answers to questions, such as “How much did you pay for the medical expense last month?” and “How much did you spend on the disease-related expenses other than medical expenses?”, were often used to measure the objective financial toxicity during cancer treatment and care [25]. Catastrophic health expenditure was defined as “when previous one year patient households’ out-of-pocket expenditure for cancer care exceeded 10% of total annual household income” [40]. One study applied a pre-existing generic financial assessment instrument, namely the PFW scale, which consists of five items on the psychosocial, two items on financial resources and one item on coping strategies [38]. One study utilised the Chinese version of the cancer-specific comprehensive needs assessment tool (CNAT) [41]. One-fifth of the studies obtained financial information through hospital billing systems [27–29, 31, 33, 42, 52].

Three instruments were used in six studies to measure the health-related quality of life (HRQoL) of cancer patients in general and disease-specific aspects of life [23, 25, 26, 35, 36, 38]. The most frequently used HRQoL instrument was the Functional Assessment of Cancer Therapy (FACT). In particular, the FACT is a two-part instrument that assesses general HRQoL related to cancer and cancer therapy (FACT-G) and tumour-specific measures, such as prostate (FACT-P).

Another instrument that was often used in the assessment of HRQoL in cancer patients was the European Quality of Life Five Dimension (EuroQol/EQ-5D), which measured well-being in five dimensions: usual activities, self-care, pain/discomfort, anxiety/depression and mobility [23, 26]. The EuroQol/EQ-5D combines self-assessment with a valuation of quality of life in which full health is scored at “one” and death at “zero”. Two studies used the European Organisation for Research and Treatment of Cancer Quality of Life Questionnaire (EORTC QLQ-C30), which consists of 30 core items with five functional scales (cognitive, emotional, physical, role and social), three symptom scales (fatigue, pain and vomiting/nausea) and a global health and quality-of-life scale [26, 35].

### Quality assessment

Supplementary Fig. 1 presents the results of the quality assessment of the included quantitative studies. Sixteen studies achieved an overall low risk of bias. Fifteen of the quantitative studies were mainly rated low on overall quality. Thirteen of the quantitative studies were rated as a moderate risk of bias often because there were no identification of potential confounders, evidence of strategies to deal with effects of confounding factors and/or description of statistical adjustment in data. Overall, two of the quantitative studies were rated as a high risk of bias because of outcome measurement and statistical analysis issues. Outcome measurement issues were due to the use of unvalidated instruments and lack of clear definition and documentation of outcomes. The qualitative study demonstrated low risk of bias. It showed sufficient quality in terms of underlying research method, data collection and analysis [53].

## Discussion

This systematic review and meta-analysis describes the prevalence of cancer-related financial toxicity, its determinants and how it has been measured in LMICs based on available data published from 2007 to 2020. The prevalence of objective financial toxicity among cancer patients in LMICs varied significantly, ranging from 17.73 to 93.38%. There are several direct medical costs, direct non-medical costs and indirect costs that have an impact on cancer patients, their families and friends. For instance, the mean direct medical costs per cancer patients were $2740.18 and the costs attributable to surgery, radiotherapy, chemotherapy, hormone therapy and palliative care were $1678.80, $4131.50, $6555.98, $1471.27 and $3741.28, respectively. Direct non-medical costs, which included disease-related transfer, accommodation and informal and transportation costs, were hardly measured in the studies reviewed. Similarly, there is limited knowledge when it comes to measuring subjective financial toxicity and included studies scarcely focused on it. This finding confirms previous observation that there is a lack of accepted definition of subjective financial toxicity [55].

The review shows the frequent use of unvalidated or unreliable instruments for measuring financial toxicity among cancer patients in LMICs. Unvalidated instruments may generate data that do not contribute to a better understanding of cancer patients’ financial difficulties because that data cannot be interpreted effectively. Similar results have been reported by a previous systematic review, which synthesised methods for measuring financial toxicity after cancer diagnosis with most of the included studies conducted in HICs, such as the USA and UK [8]. Few standardised instruments have been developed and validated in an attempt to quantify financial toxicity in cancer patients. Examples of such instruments include Breast Cancer Finances Survey Inventory [56], PFW Scale [11] and COST [10, 57]. These tools were developed in HICs and available mostly in these countries where cancer patients’ experience of financial toxicity differs from their counterparts in LMICs. Thus, there is a need to develop a simple and cost-effective instrument that is applicable to LMICs.

The limited data in this study does not show clear evidence that health insurance is a determinant of financial toxicity. Data from six studies did not reach statistical significance [23, 38, 43–46]. However, the inclusion of data from China may, in part, explain this [44]. A recent study has demonstrated that government’s health insurance coverage significantly increased utilisation of expensive targeted anti-cancer medicines and improved patient’s affordability [58]. Despite the insufficient data to examine the relationship between health insurance and financial toxicity, it is critical to implement strategies to make health insurance systems sustainable and facilitate access to affordable cancer treatment and care. Previous studies have also highlighted that rural dwellers are less likely to access cancer treatment and care due to the lack of health insurance, travel distance and financial burden [59, 60]. Innovative strategies, such as tele-consultation and cancer patient–assisted travel schemes, can be implemented to reduce rural–urban health inequities by decreasing out-of-pocket costs.

The review shows that household size of more than four, multiple cycles of chemotherapy and private health facilities are significantly associated with objective financial toxicity. It is well known that cancer drugs remain unaffordable in most LMICs, with a large number of cancer patients delaying or skipping treatment resulting in decreased quality of life. To prevent the potential financial and clinical harms, it is critical to provide cost-effective cancer care by reducing overuse of anti-neoplastic medication [61]. Also, cancer patient groups, health professionals and governments can engage pharmaceutical companies to implement policies or interventions to lower the cost of cancer drugs. The association between large household size and objective financial toxicity is consistent with the literature on financial toxicity in traumatic injury [62]. Large household size in most LMICs can be explained by the high infant mortality that translates into insecurity in families about the survival of their children [63]. Previous studies have indicated that a large number of children result in the decline of parents’ participation in the labour force [64]. It also reduces household savings which exposes larger families to income shortfalls. Long-term, community ownership, community-led partnership and results-based interventions must be considered to ensure sustainable development, poverty and child mortality reduction in LMICs.

The results from this systematic review and meta-analysis support previous systematic reviews [65–67] and individual studies [7, 9] showing that adult patients with newly diagnosed cancer experience significantly objective financial toxicity and impaired HRQoL. It is important to note that the deteriorating HRQoL occurred in several domains, including physical well-being, social well-being, emotional well-being and functional well-being. As demonstrated by a study from HIC, financial toxicity directly impacts the complete well-being of gastrointestinal cancer patients with higher earners reporting less challenges with accessing community resources, pain, fatigue, anxiety and depression [68].

The results of this study show that cancer patients in LMICs often need to finance their medical and non-medical costs by using personal savings, selling assets, skipping bill payments, borrowing or incurring bank debt. Waiving medical bills and implementing social policies that assist with necessities, such as food, accommodation and transport for treatment are critical coping strategies to reduce the financial impact on cancer patients and their families. However, previous studies [69, 70] have reported that most African countries have limited or no social protection systems to provide safety nets for patients, thereby forcing unsustainable coping strategies that increase the risk of bankruptcy.

### Strengths and limitations

Strengths of this study include the comprehensive search strategies, rigorous selection criteria and a thorough review process. This is the first systematic review and meta-analysis to identify the extent of cancer-related financial toxicity and how it has been measured in LMICs. There are limitations in this study. First, substantial heterogeneity in the included studies was detected. Hence, we applied random-effects model, which allows for the true effect to vary between studies. We also used sub-group analysis to help with the interpretation of results. Second, it was challenging to explicitly model cost variables and determinants in the meta-analysis due to several reasons, including incomplete reporting and the limited number of included studies.

## Conclusion

This systematic review and meta-analysis indicate that cancer diagnosis, treatment and care impose high financial toxicity on cancer patients in LMICs. More high-quality research on cancer-related financial toxicity is needed, particularly from Africa. Future research needs to create and validate an instrument that will be available to LMICs to measure financial toxicity in cancer patients.

## Supplementary Information

Below is the link to the electronic supplementary material.

Supplementary file1 (DOCX 46 KB)

Supplementary file2 (DOCX 4186 KB)

## Data Availability

All data generated or analysed during this study are included in this published article.

## Code availability

N/A.
