# Supplementary material for: Financial toxicity of cancer care in low- and middle-income countries: a systematic review and meta-analysis
Source: Support Care Cancer. 2022 Apr 25;30(9):7159–90. doi: 10.1007/s00520-022-07044-z (PMC9385791; doi:10.1007/s00520-022-07044-z)
Supplement: Supplementary file 1 — Supplementary file1 (DOCX 46 KB) [file 520_2022_7044_MOESM1_ESM.docx]

**Journal:** Supportive Care in Cancer

**Title:** Financial toxicity of cancer care in low and middle-income countries: a systematic review and meta-analysis

# Authors

Corresponding author:

1. **Andrew Donkor** – Improving Palliative, Aged and Chronic Care through Clinical Research and Translation (IMPACCT), Faculty of Health, University of Technology Sydney, Sydney, New South Wales, Australia

Department of Medical Diagnostics, Faculty of Health Sciences, Kwame Nkrumah University of Science and Technology, Ghana

Email: [Andrew.Donkor@uts.edu.au](mailto:Andrew.Donkor@uts.edu.au)

ORCID ID: 0000-0002-6073-524X

All other authors:

1. **Vivian Della Atuwo-Ampoh** – Department of Medical Imaging, School of Allied Health Sciences, University of Health and Allied Sciences, Ho, Ghana

Email: [vdatuwo-ampoh@uhas.edu.gh](mailto:vdatuwo-ampoh@uhas.edu.gh)

1. **Frederick Yakanu** – National Centre for Radiotherapy, Korle-Bu Teaching Hospital, Accra, Ghana

Email: [f.yakanu@gmail.com](mailto:f.yakanu@gmail.com)

1. **Eric Torgbenu** – Improving Palliative, Aged and Chronic Care through Clinical Research and Translation (IMPACCT), Faculty of Health, University of Technology Sydney, Sydney, New South Wales, Australia; Department of Physiotherapy and Rehabilitation Sciences, University of Health and Allied Sciences, Ho, Ghana

Email: [eric.l.torgbenu@student.uts.edu.au](mailto:eric.l.torgbenu@student.uts.edu.au)

ORCID ID: 0000-0002-1065-1718

1. **Edward Kwabena Ameyaw** – The Australian Centre for Public and Population Health Research (ACPPHR), Faculty of Health, University of Technology Sydney, Sydney, New South Wales, Australia

Email: [edward.k.ameyaw@student.uts.edu.au](mailto:edward.k.ameyaw@student.uts.edu.au)

ORCID ID: 0000-0002-6617-237X

1. **Doris Kitson-Mills –** Department of Radiography, University of Ghana, Accra, Ghana

Email: [doriskitsonmills@gmail.com](mailto:doriskitsonmills@gmail.com)

1. **Verna Vanderpuye** – National Centre for Radiotherapy, Korle-Bu Teaching Hospital, Accra, Ghana

Email: [vanaglat@yahoo.com](mailto:vanaglat@yahoo.com)

ORCID ID: 0000-0003-3656-6965

1. **Kofi Adesi Kyei –** Department of Radiography, University of Ghana, Accra, Ghana

Email: [kakyei@ug.edu.gh](mailto:kakyei@ug.edu.gh)

ORCID ID: 0000-0003-3485-5368

1. **Samuel Anim-Sampong –** Department of Radiography, University of Ghana Accra, Ghana

Email: [tychicus77@gmail.com](mailto:tychicus77@gmail.com)

1. **Jamal Khader** – Radiation Oncology Department, King Hussein Cancer Centre, Amman, Jordan

Email: [jkhader@khcc.jo](mailto:jkhader@khcc.jo)

1. **Omar Khader** – Faculty of Medicine, University of Jordan, Amman, Jordan

Email: [omar.j.khader@gmail.com](mailto:omar.j.khader@gmail.com)

**Supplementary Table 1: Search strategy for the Ovid MEDLINE**

| #1 | exp Neoplasms/ |
| --- | --- |
| #2 | (cancer or tumo* or malignan*).ti,ab |
| #3 | exp "Delivery of Health Care"/ or exp diagnosis/ or exp therapeutics/ or exp radiotherapy/ or exp Specialties, Surgical/ or palliative care/ or terminal care/ or hospice care/ or survivorship/ |
| #4 | exp Cancer Survivors/ or exp Survivors/ |
| #5 | (“cancer survivor*” or patient or survivor or sufferer or family or relative).ti,ab. |
|  | exp "Cost of Illness"/ or exp "Costs and Cost Analysis"/ |
| #6 | (cost or productivity loss or expense or bill* or “cost of illness” or treatment cost or ‘out-of-pocket’ or economic burden or financial toxicity or financial hardship or financial burden or financial effect or financial risk or financial stress or financial distress or copayment or deductible).ti,ab. |
| #7 | exp “quality of life”/ or exp mortality/ or exp morbidity/ or exp “burden of illness”/ |
| #8 | (outcome or life or well-being or effect or health or survival or stress or mental health or impact) |
| #9 | exp developing country/ |
| #10 | (Afghanistan or Benin or ‘Burkina Faso or Burundi’ or Central African Republic or Chad or Comoros or Democratic Republic of the Congo or Eritrea or Ethiopia or Gambia or Guinea or Guinea-Bissau or Democratic People's Republic of Korea or Liberia or Madagascar or Malawi or Mali or Mozambique or Nepal or Niger or Rwanda or Senegal or Sierra Leone or Somalia or South Sudan or Tanzania or Togo or Uganda or Zimbabwe or Armenia or Bangladesh or Bhutan or Bolivia or Cape Verde or Cambodia or Cameroon or Congo or Cote d'Ivoire or Djibouti or Egypt or El Salvador or Ghana or Guatemala or Honduras or India or Indonesia or Kenya or Kosovo or Kyrgyzstan or Lesotho or Mauritania or Micronesia or Moldova or Mongolia or Morocco or Myanmar or Nicaragua or Nigeria or Pakistan or Papua New Guinea or Philippines or Samoa or Sri Lanka or Sudan or Swaziland or Syria or Tajikistan or Timor-Leste or Tonga or Tunisia or Ukraine or Uzbekistan or Vanuatu or Vietnam or Yemen or Zambia or Albania or Algeria or American Samoa or Angola or Argentina or Azerbaijan or Republic of Belarus or Belize or “Bosnia and Herzegovina” or Botswana or Brazil or Bulgaria or Colombia or China or Costa Rica or Cuba or Dominica or Dominican Republic or Equatorial Guinea or Ecuador or Fiji or Gabon or Georgia or Grenada or Iran or Guyana or Iraq or Jamaica or Jordan or Kazakhstan or Lebanon or Libya or Macedonia Republic or Macedonia Republic or Malaysia or Mauritius or Mexico or Montenegro or Namibia or Palau or Panama or Paraguay or Peru or Romania or Russia or Serbia or South Africa or Saint Lucia or “Saint Vincent and the Grenadines” or Suriname or Thailand or Turkey or Turkmenistan or Venezuela) |
| #11 | (third world countr* or third world countr* or less-developed countr* or less-developed countr* or under developed countr* or under developed countr* or underdeveloped countr* or underdeveloped countr* or developing nation* or developing nation* or less developed nation* or less developed nation* or third world nation* or third world nation* or underdeveloped nation* or underdeveloped nation* or developing countr* or developing countr* or low income countr* or low income countr* or middle income countr* or middle income countr* or middle income nation* or middle income nation*).ti,ab. |
