# Supplementary material for: Financial toxicity of cancer care in low- and middle-income countries: a systematic review and meta-analysis
Source: Support Care Cancer. 2022 Apr 25;30(9):7159–90. doi: 10.1007/s00520-022-07044-z (PMC9385791; doi:10.1007/s00520-022-07044-z)
Supplement: Supplementary file 2 — Supplementary file2 (DOCX 4186 KB) [file 520_2022_7044_MOESM2_ESM.docx]

**Journal:** Supportive Care in Cancer

**Title:** Financial toxicity of cancer care in low and middle-income countries: a systematic review and meta-analysis

# Authors

Corresponding author:

1. **Andrew Donkor** – Improving Palliative, Aged and Chronic Care through Clinical Research and Translation (IMPACCT), Faculty of Health, University of Technology Sydney, Sydney, New South Wales, Australia

Department of Medical Diagnostics, Faculty of Health Sciences, Kwame Nkrumah University of Science and Technology, Ghana

Email: [Andrew.Donkor@uts.edu.au](mailto:Andrew.Donkor@uts.edu.au)

ORCID ID: 0000-0002-6073-524X

All other authors:

1. **Vivian Della Atuwo-Ampoh** – Department of Medical Imaging, School of Allied Health Sciences, University of Health and Allied Sciences, Ho, Ghana

Email: [vdatuwo-ampoh@uhas.edu.gh](mailto:vdatuwo-ampoh@uhas.edu.gh)

1. **Frederick Yakanu** – National Centre for Radiotherapy, Korle-Bu Teaching Hospital, Accra, Ghana

Email: [f.yakanu@gmail.com](mailto:f.yakanu@gmail.com)

1. **Eric Torgbenu** – Improving Palliative, Aged and Chronic Care through Clinical Research and Translation (IMPACCT), Faculty of Health, University of Technology Sydney, Sydney, New South Wales, Australia; Department of Physiotherapy and Rehabilitation Sciences, University of Health and Allied Sciences, Ho, Ghana

Email: [eric.l.torgbenu@student.uts.edu.au](mailto:eric.l.torgbenu@student.uts.edu.au)

ORCID ID: 0000-0002-1065-1718

1. **Edward Kwabena Ameyaw** – The Australian Centre for Public and Population Health Research (ACPPHR), Faculty of Health, University of Technology Sydney, Sydney, New South Wales, Australia

Email: [edward.k.ameyaw@student.uts.edu.au](mailto:edward.k.ameyaw@student.uts.edu.au)

ORCID ID: 0000-0002-6617-237X

1. **Doris Kitson-Mills –** Department of Radiography, University of Ghana, Accra, Ghana

Email: [doriskitsonmills@gmail.com](mailto:doriskitsonmills@gmail.com)

1. **Verna Vanderpuye** – National Centre for Radiotherapy, Korle-Bu Teaching Hospital, Accra, Ghana

Email: [vanaglat@yahoo.com](mailto:vanaglat@yahoo.com)

ORCID ID: 0000-0003-3656-6965

1. **Kofi Adesi Kyei –** Department of Radiography, University of Ghana, Accra, Ghana

Email: [kakyei@ug.edu.gh](mailto:kakyei@ug.edu.gh)

ORCID ID: 0000-0003-3485-5368

1. **Samuel Anim-Sampong –** Department of Radiography, University of Ghana Accra, Ghana

Email: [tychicus77@gmail.com](mailto:tychicus77@gmail.com)

1. **Jamal Khader** – Radiation Oncology Department, King Hussein Cancer Centre, Amman, Jordan

Email: [jkhader@khcc.jo](mailto:jkhader@khcc.jo)

1. **Omar Khader** – Faculty of Medicine, University of Jordan, Amman, Jordan

Email: [omar.j.khader@gmail.com](mailto:omar.j.khader@gmail.com)

1. *Quantitative studies*





1. *Qualitative study*





**Supplementary Figure 1: Quality assessment results for the included studies**
